# Evidence for a shape-based recognition of odorants *in vivo* in the human nose from an analysis of the molecular mechanism of lily-of-the-valley odorants detection in the Lilial and Bourgeonal family using the C/Si/Ge/Sn switch strategy

**DOI:** 10.1371/journal.pone.0182147

**Published:** 2017-08-01

**Authors:** Steffen Wolf, Lian Gelis, Steffen Dörrich, Hanns Hatt, Philip Kraft

**Affiliations:** 1 Department of Biophysics, CAS-MPG Partner Institute for Computational Biology, Key Laboratory of Computational Biology, Shanghai Institutes for Biological Sciences, Chinese Academy of Sciences, Shanghai, P.R. China; 2 Department of Biophysics, Ruhr-University Bochum, Bochum, Germany; 3 Department of Cellphysiology, Ruhr-University Bochum, Bochum, Germany; 4 Institute of Inorganic Chemistry, University of Würzburg, Würzburg, Germany; 5 Fragrance Research, Givaudan Schweiz AG, Dübendorf, Switzerland; University of Leeds, UNITED KINGDOM

## Abstract

We performed an analysis of possible mechanisms of ligand recognition in the human nose. The analysis is based on *in vivo* odor threshold determination and *in vitro* Ca^2+^ imaging assays with a C/Si/Ge/Sn switch strategy applied to the compounds Lilial and Bourgeonal, to differentiate between different molecular mechanisms of odorant detection. Our results suggest that odorant detection under threshold conditions is mainly based on the molecular shape, i.e. the van der Waals surface, and electrostatics of the odorants. Furthermore, we show that a single olfactory receptor type is responsible for odor detection of Bourgeonal at the threshold level in humans *in vivo*. Carrying out a QM analysis of vibrational energies contained in the odorants, there is no evidence for a vibration-based recognition.

## Introduction

Understanding the molecular mechanisms underlying the human sense of smell is still challenging. Olfactory receptors (ORs) belong to the protein family of G protein-coupled receptors (GPCRs), which forms the largest superfamily of proteins in the human genome [1], and with nearly 350 functional genes of ORs out of ca. 1000 OR genes in total [2], olfactory receptors in turn form the largest subfamily of GPCRs. Besides their role as major detectors of airborne odorants, ORs recently were found to be present in a large set of different human tissues as well [3,4]. A functional role of these ectopically expressed ORs was shown in human colon tissue [5], sperm [6], blood cells [7], skin tissue [8], brain [9,10], smooth muscles [11], and melanocytes [12]. Furthermore, the expression of some ORs is up–regulated in different types of cancer cells [13–17], and these ORs have an effect on cell proliferation in at least prostate [3], liver [18], and leukemia [19] cancer cells. While they commonly are counted as members of the rhodopsin-like GPCRs [20], recent research points to them forming a separate GPCR subgroup of their own [21]. In agreement with this, olfactory receptors exhibit peculiar activation properties: they seem to exhibit an “analog” response behavior, i.e. a single OR can be activated by different small organic molecules with a broad range of chemical modifications, and in turn give a signal response level, which may depend on the respective ligand bound [22–24]. As a counterexample, rhodopsin [25,26], the light-activated receptor in human rod cells, exhibits a “digital” response behavior, i.e. forms well-defined active and inactive states, and thus clear signal response levels. The exact mechanism causing the analog response is still under debate. In general, the interaction of a ligand with its receptor counterpart is mediated by the full range of intermolecular interactions (both attractive and repulsive) that matter, resulting in both negative free energy contributions ranging from van der Waals to hydrogen bonding, cation-pi and ion-ion interactions to metal coordination (i.e., weak covalent bonding), or positive free energy contributions (e.g., steric repulsion, dipole-dipole mismatches, hydrophobicity and other solvation-entropic contributions) [27]. If we want to envision an odorant/receptor interaction in the framework of a classical Lock and Key model, we need to define a matching molecular surface or “shape” for both components. For quantum scale objects, this shape is generally given by the van der Waals radius, i.e. the distance of minimal free energy. So far, odorant recognition was proposed as being based on recognition of their chemical scaffold, including matching shape and electrostatics components (so-called “odotopes” [28,29] or “olfactophore models” with “profile” or “bulk” groups bound via their “osmophoric” functional group to a hydrogen-bond acceptor or donor [30–34]). In support of this approach, it was recently shown that a functional relationship exists between molecular volume and the olfactory neural response with the maximum affinity occurring when the molecular volume of an odorant matches the volume of the binding pocket [35], and that the odorant affinity of a receptor is based on the molecular frame of a given odorant [36]. Sensitivity for defined chemical functionalities (e.g. thiols) can be increased via the usage of metal cofactors (e.g. copper ions) by the receptor [37–40]. Alternatively, odorant recognition was proposed to be based on molecular vibrations [41–46], Furthermore, a combination of scaffold/vibration recognition [47,48], or a combination of shape recognition and matching protein–ligand dynamics [49,50] was proposed to result in olfactory receptor activation.

In this article, we aim to gain insight into the molecular details of ligand interaction with ORs both *in vitro* and *in vivo*. It is necessary to discriminate between the different processes taking place between the stages of physical uptake of an odorant into the body and the final physiological response: in this article, we will focus on the process of odor detection, which is the uptake of an odorant by an olfactory receptor, and the subsequent activation of the latter by the former. However, to gain insight into this process directly within the human nose, we will use the quantitative perception of human individuals as a readout parameter, i.e. the process of an individual cognitively detecting the presence of an odorant, without detailing on the odor quality. Odor thresholds, thus, are the quantitative limits of odor concentration detection. Opposed to that, odor qualitative perception is the assignment of a odor quality (such as floral, fruity, wooden etc.), which is encoded by specific combinations of activated olfactory receptor cells in the olfactory epithelium (OE) that results in a spatiotemporal pattern of glomerular activation in the olfactory bulb [51]. Major insights on these mechanism of olfactory information processing in mammals result from experiments on rodents: here, each type of olfactory cell carries a single OR type [52,53]. Glomerular activation increases with odor concentration [54]: at threshold concentration, only a single or very few glomeruli are activated, this activation is necessary for odor detection at these particular concentrations. With increasing odor concentrations new glomeruli are recruited [51,54–63]. As we evidently cannot perform such invasive experiments with humans, we here rely on a theoretical analysis of quantitative perception experiments with humans to gain insight into the process of odorant detection on the molecular level. We have to state that the connection between these two effects may contain non-linear effects during periperception [64,65], e.g. odorant pre-binding to the nasal mucus or odorant-binding proteins [66,67], and signal processing in the olfactory neuronal cells, the olfactory bulb, or the human brain. However, as the molecular perturbations introduced to the investigated odorants are small, as we will detail on in the following, we assume them to only affect receptor binding. Furthermore, as we rely on the computation of changes of quantitative perception relative to these perturbations, we assume all absolute errors due to non-linearity effects between odorant detection on the molecular level and the physiological effect of quantitative perception to cancel out, so that in the end, we can use data on physiological perception to make a statement on the molecular detection basis directly at the receptor level *in vivo*.

**Fig 1:**
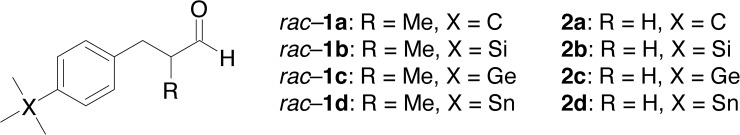
Chemical structures of the Lilial 1a–1d and Bourgeonal 2a–2d derivatives investigated.

In order to further elucidate the molecular mechanism of odorant recognition in humans, we recently published the *in vitro* and *in vivo* results of a strategic sila-, germa-, and stanna-substitution of the quaternary carbon atom in the hydrophobic bulk group of the lily-of-the-valley odorants Lilial (**1a** → **1b**/**1c**/**1d**; compounds studied as racemates) and Bourgeonal (**2a** → **2b**/**2c**/**2d**) (see Fig 1) [68–71]. The advantage of this strategy is that this type of substitution leads to model compounds with the same molecular geometry, but steadily increasing hydrophobic bulk group size, and thus only introduce a small perturbation of the overall molecular shape, which should only affect receptor/ligand binding, but not the overall physicochemical properties of the compound set, and therefore not cause any differences in putative periperception effects or alter the activation properties towards the receptor. The study was carried out with the molecular scaffolds of the lily-of-the-valley odorants Lilial (**1a**) and Bourgeonal (**2a**), and hOR17-4 (gene name: hOR1D2) as the detecting receptor [6,69].

We now present complementary computational and theoretical studies on odor detection by hOR17-4, which we currently assume to be the most sensitive receptor for **1a–1d** and **2a–2d** detection in the human nose [69,71], as well. We investigate the molecular mechanism of odorant recognition, with a special focus on the *in vivo* data set from our earlier works [71]. Carrying out quantum mechanics (QM) calculations on the molecular geometries and vibrations of **1a–1d** and **2a–2d**, docking into the binding site of a model of hOR17-4, and structure–activity relationship (SAR) analyses [69,71] by comparing the *in vivo* data with predictions from statistical mechanics, we find that the process of odor detection *in vivo* most likely relies on a single receptor type only, that this process is indeed based on ligand shape interactions and electrostatics, and does not contain any dependence on odorant vibrations.

## Materials and methods

DFT calculations on **1a**–**1d** and **2a**–**2d** were performed with Gaussian09 [72]. For calculations on the carbon, silicon, and germanium compound geometries and atomic charges, B3LYP/6-311G** was used [73], while for tin compounds, the LANL2DZ pseudopotential was used for the tin atom and 6-311G** for the rest of the molecule [74]. Molecule conformations were initially minimized in vacuum. Atomic charges were then calculated with the ESP method [75]. To follow up on the docking studies reported earlier [69], a static protein model was build according to ref. [76] based on the hOR17-4/rhodopsin alignment proposed earlier [69]. Please note that as the experimental investigations [69,71] used a racemic mixture of compounds **1a**–**1d**, so it was necessary to perform docking with both enantiomers of **1a**–**1d**.

For Docking, ligand topologies for (*R*)-**1a**–(*R*)-**1d**, (*S*)-**1a**–(*S*)-**1d**, and **2a**–**2d** were obtained from the PRODRG server [77], with atomic charges from ESP charge calculations mentioned above. For describing the van der Waals radii of the heteroatoms, we used the van der Waals radius of carbon. We are aware that this might induce a small bias, but refrained from creating new heteroatom van der Waals parameters, as this type of interaction is very hard to parameterize in an appropriate way. However, the major effect of the heteroatom replacement, besides change of charges, is the elongation of the X–C bond and the resulting increase of hydrophobic bulk group size. As the heteroatom is located in the core of this bulk group and surrounded by methyl groups, its own van der Waals sphere is mostly shielded. We rate the resulting error in van der Waals interaction as small enough to be neglected. Docking was carried out with Autodock Vina [78].

**Fig 2:**
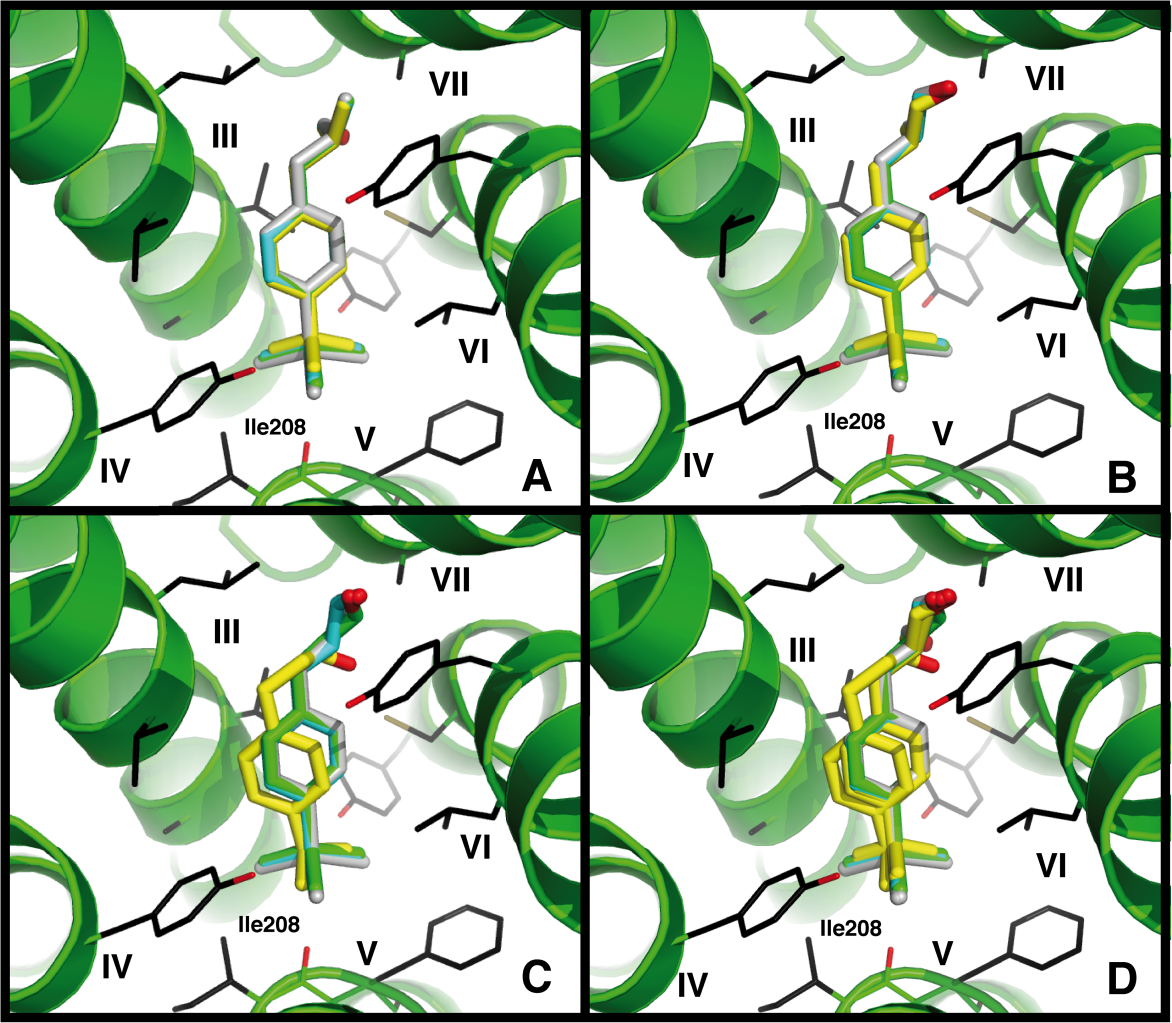
Docking poses of 1a–1d and 2a–2d at hOR17-4. (A) (*S*)-**1a**–(*S*)-**1d**. (B) (*R*)-**1a**–(*R*)-**1d**, (C) **2a**–**2d**, (D) **1a**–**1d** and **2a**–**2d**. Carbon compounds (**1a** and **2a**) are shown in yellow, silicon compounds (**1b** and **2b**) in cyan, germanium compounds (**1c** and **2c**) in green, and tin compounds (**1d** and **2d**) in grey. Helices are numbered in Roman numerals. All docking poses share a high similarity throughout the entire set of compounds. The carbonyl oxygen atom of Ile208 forms a favorable contact with the hydrophobic bulk group of the investigated ligands.

For the analysis of the vibrational energy contributions, we used ligand geometries of the best docking poses of ligands found in docking runs (as displayed in Fig 2) and all protein heavy atoms within 4 Å of the respective ligand. To avoid basis set superposition errors (BSSE), we additionally included the remaining three ligands into the calculation box at a distance of >10 Å from the protein/ligand binding site model. Missing valences were saturated by the addition of additional hydrogen atoms. All heavy atoms in the resulting QM boxes were fixed in their positions, while hydrogen atoms were allowed to move. After an initial minimization of Hartree-Fock level (basis sets as given above), followed by a minimization on B3LYP level, a normal mode analysis was carried out by calculation and diagonalization of the respective Hessian matrices for the determination of binding pocket model vibrations, the respective vibrational energies and of free energies of binding [79].

Data analysis was performed with Gnuplot [80], Origin v7.0, and MS Excel. Molecular figures were prepared with PyMOL [81].

## Results and discussion

Based on the results from the *in vivo* odor threshold determinations reported in [71], we performed an analysis of the biological data together with energies obtained from docking and QM calculations. Our major focus was on elucidating the mechanism that determines odorant detection by hOR17-4 *in vitro* and in the human nose. To shed light on the different possibilities of how *rac*-**1a**–*rac*-**1d** and **2a**–**2d** are recognized by hOR17-4, we performed a docking analysis as in earlier work [69] using a receptor structure with a static protein backbone. Docking itself is a method which deduces the ligand-binding strength only from van der Waals surface (“shape” as we define it here) and charge complementarities with the ligand binding pocket. It should therefore allow us to exclusively assess the contribution of molecular shape/steric fit and electrostatics to odorant recognition. Fig 2 shows the resulting docking poses, and Table 1 gives the respective calculated free energies of binding. As can be seen from Fig 2, all the compared poses are highly similar. The major differing observable in our QM calculations on the geometries of this set of compounds are the bond distances between heteroatoms and the methyl and phenyl moieties, respectively. To create a general distance observable characteristic for one molecule, and as the other bond lengths only vary on the order of 0.01 Å, we define an average X–C distance, which is the average of all four X–C distances, as reaction coordinate. At the same time, this distance is effectively the radius of the hydrophobic bulk group, and thus is a measure for its size, too. The resulting average X–C distances are: C–C, 1.54 Å; Si–C, 1.89 Å; Ge–C, 1.98 Å; Sn–C, 2.15 Å. As a first step, we wanted to check qualitatively how this elongation of the average X–C distance affects the binding affinity in an *in silico* docking of our ligand set, and how well it reflects the EC_50_ and maximal receptor activation (E_max_) as assessed by *in vitro* measurements [71].

**Table 1:** Docking free binding energies.

Element	(*S*)-1	ΔΔ*G*_*act/bind*_	(*R*)-1	ΔΔ*G*_*act/bind*_	2	ΔΔ*G*_*act/bind*_
a (C)	–5.6	0.4	–5.6	0.1	–6.2	0.0
b (Si)	–5.3	0.8	–5.1	1.0	–5.6	0.4
c (Ge)	–5.1	1.0	–4.9	1.2	–5.3	0.5
d (Sn)	–4.6	1.5	–4.3	1.8	–4.9	0.8

Calculated free binding energies (Δ*G*_*bind*_) in kcal mol^–1^ of binding modes of Lilial compounds (*S*)-**1a** to (*S*)-**1d**, (*R*)-**1a** to (*R*)-**1d**, and Bourgeonal compounds **2a** to **2d** displayed in Fig 2 as obtained from docking assays, and differences of Δ*G*_*bind*_ of the displayed (activation causing) and the respective best observed binding mode (ΔΔ*G*_*act/bind*_) in kcal mol^–1^.

Performing docking with Autodock Vina directly returns free binding enthalpies Δ*G*_*bind*_ as scoring values. Note that as the experimental investigations used a racemic mixture of compounds **1a**–**1d**, we needed to perform docking with both enantiomers of **1a**–**1d**, since one form might bind better to the receptor than the other. A separate experimental application of both enantiomers did not seem reasonable as they racemize very easily and rapidly via enol formation, so that one never could be sure to have the pure enantiomers at hand.

The calculated Δ*G*_*bind*_ values (Table 1) for the respective activating docking poses show a continuation of the trends observed for the C/Si pairs *rac*-**1a**/*rac*-**1b** and **2a**/**2b** reported earlier [69]: With increasing atomic number (C → Si → Ge → Sn), binding becomes less and less favorable. This is in agreement with the apparent shift of *in vitro* EC_50_ to higher values for compounds *rac*-**1a**–*rac*-**1d** and **2a**–**2d** [71]. Interestingly, the docking poses shown in Fig 2 resemble those reported earlier for *rac*-**1a**, *rac*-**1b**, **2a**, and **2b** [69], but are rotated by 180˚ in comparison to them. In our docking approach, we only observed this particular binding mode for **2a** with a free binding affinity of –5.5 kcal mol^–1^, which is 0.7 kcal mol^–1^ higher than that found for the best docking pose. Earlier works [69] were based on a manual docking approach. In this work now, we employ an automatic docking approach, which should not be biased by human perception and expectation. Compound **2a** is the best binder and activator in the *in vitro* experiments; therefore, we defined its best binding posture as receptor activation binding mode. For comparison, we checked the binding energies of the other ligands in the same docking position and orientation. In our docking assay, the similar free binding enthalpies of (*R*)-**1a** and (*S*)-**1a** (both –5.6 kcal mol^–1^) and **2a** (–6.2 kcal mol^–1^) are in relatively good agreement with the experimental observation that *rac*-**1a** and **2a** exhibit comparable *in vitro* EC_50_ values (125 μM and 130 μM, respectively) [71]. However, it should be noted that the calculated energy differences are quite small, with a maximal difference of 1.4 kcal mol^–1^, which might easily be affected by the thermal fluctuation of the amino acid side chains defining the odorant binding pocket. Concerning the *in vitro* maximal activation potency *E*_max_ values, we assume that the loss in activation potency with increasing atomic number (Fig 2, ref. [71]) of both *rac*-**1a**–*rac*-**1d** and **2a**–**2d** derives from the presence of other binding postures of the respective ligands at the receptor, both within or close to the proposed orthosteric binding site. Such alternative binding postures will lead to ligand binding, but not to subsequent receptor activation, similar to the effect of an antagonist. If these alternative positions are energetically more favorable than the receptor activating binding pose, they will be occupied more often than the activating binding pose. An increase in the energetic difference between the activation causing binding mode and the energetically best binding mode (ΔΔ*G*_*act/bind*_) will lower the experimentally observed *E*_*max*_. We therefore checked if the activating binding mode was the energetically most favorable binding mode. If it was not, we analyzed the resulting ΔΔ*G*_*act/bind*_. The results are displayed in Table 1: for compounds **2a**–**2d** we observed that with increasing atomic number of the heteroatom, ΔΔ*G*_*act/bind*_ indeed increased stepwise with a maximum of 0.8 kcal mol^–1^ for **2d**, which is in good agreement with the stepwise decline in *E*_max_ observed *in vitro* (Fig 2, ref. [71]). In the case of Lilial-based compounds, the carbon compounds (*S*)-**1a** and (*R*)-**1a** both already exhibit an ΔΔ*G*_*act/bind*_ value different from zero ((*S*)-**1a**: 0.4 kcal mol^–1^; (*R*)-**1a**: 0.1 kcal mol^–1^). As the experimental investigation we refer to used a racemic mix of both compounds, (*S*)-**1a** and (*R*)-**1a** are always present together. Therefore, the best binding pose of (*S*)-**1a** can interfere with the receptor activating binding pose of (*R*)-**1a**, and vice versa. The receptor activating postures of (*S*)-**1a** and (*R*)-**1a** exhibit the same Δ*G*_*bind*_ (–5.6 kcal mol^–1^). In this case, we need to search for the compound with largest ΔΔ*G*_*act/bind*_ value to determine the best binding compound, which is (*S*)-**1a** with a ΔΔ*G*_*act/bind*_ of 0.4 kcal mol^–1^. As stated before, this value applies for both enantiomers, as (*S*)-**1a** will block the binding site for (*R*)-**1a**, too. This is in good agreement with the observation from *in vitro* experiments that *rac*-**1a** only exhibits 50% of the *E*_max_ of **2a**. Furthermore, the atomic number dependent increase of ΔΔ*G*_*act/bind*_ for (*S*)-**1a**–**1d** and (*R*)-**1a**–**1d** is larger than the one of compounds **2a**–**2d**, with a maximum of 1.8 kcal mol^–1^ for (*R*)-**1d** 0.8 kcal mol^–1^ for **2d**. This is in good agreement with *rac*-**1b**–**1d** effectively being inactive compounds in our *in vitro* experiments, while **2c** and **2d** show residual activity. The reason for this presence of alternative binding modes in *rac*-**1a**–*rac*-**1d** seems to be a different (and most likely unfavorable) positioning of the aldehyde osmophore close to helix VII (see Fig 2), which differs from the orientation found in **2a**–**2d** due to the presence of the additional methyl group in the Lilial series. We are aware that the experimental setup contains a certain level of ambiguity, as the signal cascade between our input via ligand application and the readout via Ca^2+^ concentration changes might exhibit non-linear effects. Therefore, we do not try to perform a quantitative analysis. However, we observe a clear qualitative agreement between the increase in average X–C distance and the increase in experimental EC_50_ and the decrease of *E*_max_ values. Because of this agreement, we conclude that hOR17-4 recognizes our set of odorants by a shape- and electrostatics-based recognition.

At this point, we have to state that it is ultimately the free energy of binding that is correlated with odor perception. The X–C distance serves only as a reaction coordinate, but is not the main reason for the changes in Δ*G*_*bind*_ within compounds *rac*-**1b**–**1d** or **2a**–**2d**. However, as stated above, the only differences the two ligands exhibit are a) the different X–C distances, and b) the atomic charges on the X atom and the methyl groups surrounding it. The increased X-C distance results in an increased van der Waals radius of the terminal hydrophobic bulk group, as well, increasing the ligand van der Waals surface, and thus a change in “shape”, as we define it. In this case, the X–C reaction coordinate is indicative for both an increase in ligand volume and a change in its electrostatics, which are the only varying parameters to determine the binding free energy.

In order to quantify the contribution of this shape-dependent ligand recognition, we investigated the nature of the interaction of *rac*-**1a**–*rac*-**1d** and **2a**–**2d** with odorant receptors in the human nose *in vivo*. For this we focused on the dependence of the experimental odor thresholds from the average X–C distance *r* in the XMe_3_ groups, which we obtained from QM calculations.

If different receptor types are involved together in odorant detection of our set of odorants, they all possess different ligand-binding cavities, and all receptors should be affected differently by the C/Si, C/Ge, and C/Sn exchange in their activation behavior. As mentioned above, this X–C distance *r* we herein refer to is the average overall X–C distance found in the full ligand, i.e. the average of one time the X–phenyl ring distance, and three times the X–methyl group distance. If we assume the hydrophilic bulk group to roughly exhibit the form of a sphere, then this average X–C distance is equal to the radius of this sphere. With our heteroatom replacement scheme, we are able to manipulate this radius stepwise with a high spatial resolution from 1.54 Å to 2.15 Å. This means that we can manipulate the bulk group diameter, and therefore the overall size of the bound ligands, over a range of 1.22 Å (equal to 2 × 0.61 Å, which is the difference between 1.54 Å and 2.15 Å). An analysis of GPCR crystal structures of beta-adrenergic receptors in active and inactive states revealed that the ligand-regulated distance of helices V and VII only differed by about 1.3 Å between both states [82,83]. Transferring this fact to our investigated system, we should be capable to almost cover the full distance range of ligand sizes between receptor-activating and inactive compounds, basing on the same ligand scaffold. As we could see in the section above, the elongation of the X–C distance *r* mostly affects Δ*G*_*bind*_, and thus the association constant *K*_a_:

$$K_{\text{a}}=e^{-\frac{\Delta G_{bind}}{\text{R}T}}$$ (1)

If the receptor activation depends on ligand binding only, and thus adheres to the laws of statistical mechanics only, the odor threshold concentration [*O*] is directly coupled to the ligand binding to the receptor, and thus to *K*_a_. If the heteroatom exchange only introduces a small perturbation into the whole ligand shape (as mentioned above), we can assume that Δ*G*_*bind*_ depends linearly on r. Following Eq (1), *r* and [*O*] can therefore be connected via an exponential function of the form

$$\left[O\right]=A\cdot e^{B\cdot r}$$ (2)

with two fit variables *A* and *B*. In this equation, *A* has the unit of ng L^–1^, while *B* is given in Å^–1^.

If multiple receptors contribute to odor detection, we assume the signaling of all involved receptors to add up to an overall response signal of all receptors involved. This is in agreement with the theory that glomeruli are sequentially recruited, i.e. the number of glomeruli that are active increases with increasing concentration of the odorant stimulus [84]. Thus, [*O*] should be connected with *r* by a summation of exponential functions

$$\left[O\right]=A\cdot e^{B\cdot r}+C\cdot e^{D\cdot r}+\ldots$$ (3)

with as many exponential functions as receptors are involved. However, as we are restricted to four data points, the usage of more than one exponential function would be statistically invalid. We therefore investigated if one exponential function is sufficient to describe the connection between [*O*] and the average X–C distance *r*, or if more functions are necessary. This corresponds to the question / Null hypothesis: is one receptor type sufficient to perform lily-of-the-valley odor detection *in vivo*?

**Fig 3:**
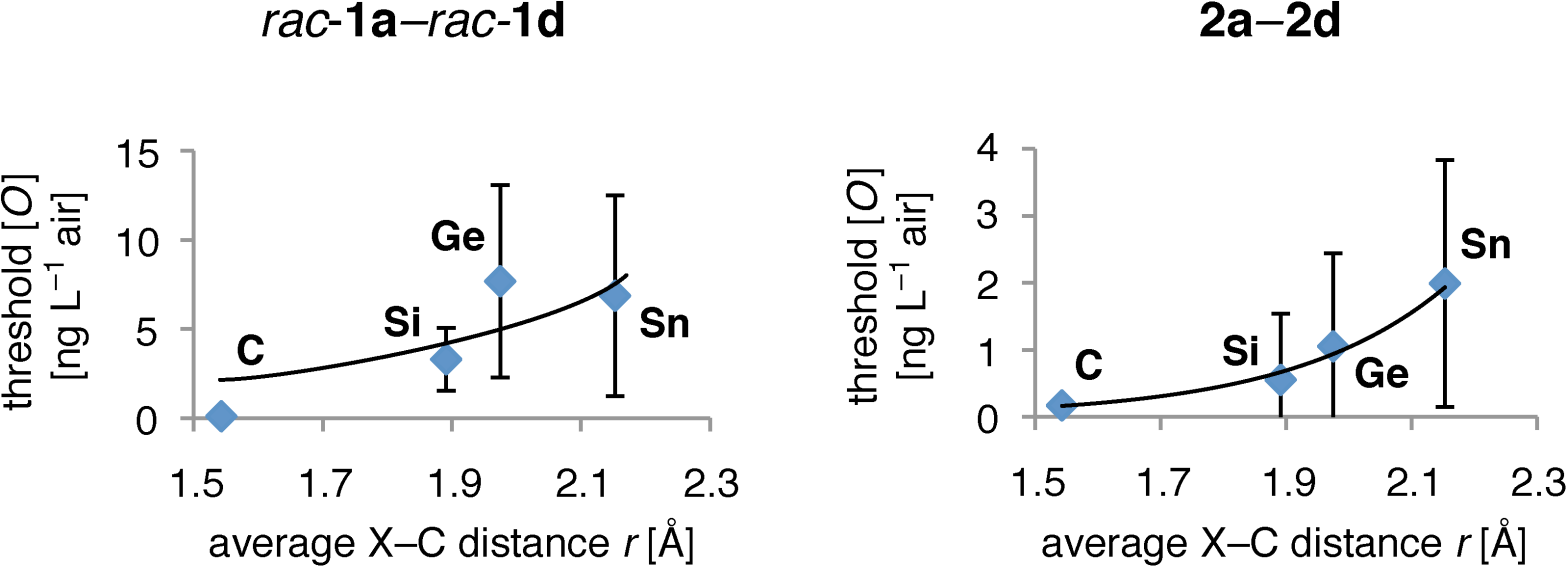
Exponential regression of odor threshold concentration [*O*] and average X–C distance *r* (X = C, Si, Ge, Sn). *In vivo* data points [71] are given as blue diamonds, regression curves as black lines. Error bars depict the standard deviation. The data points of *rac*-**1a**–*rac*-**1d** deviate from an exponential form, while the data for **2a**–**2d** follow an exponential form.

Fig 3 shows the result of this analysis. The data points for *rac*-**1a**–*rac*-**1d** deviate from an exponential curve, while the data for **2a**–**2d** follow a nice exponential form. This result agrees with the values presented in Table 2: *rac*-**1a**–*rac*-**1d** exhibit a poor R^2^ value of 0.69 value for the single exponential fit, while **2a**–**2d** exhibit an excellent R^2^ value of 0.99. It thus seems that it is a single receptor type that performs Bourgeonal detection *in vivo*, which however is not the case for Lilial detection. We are aware of the fact that the experimental data points exhibit a large standard deviation. This is due to the fact that they represent results from experiments based on human perceptions, which naturally contain a broad distribution. Furthermore, the experiments could naturally only be carried out with a small number of iterations since organolead compounds are even more reactive and toxic than the organotin derivatives **1d** and **2d**, and with a half life of 5 s (^285^Fl) transactinide organoflerovium compounds are practically inacessible. In terms of molecular weight, volatility and receptor dimensions, stanna-Lilial (**1d**, *M*r 311.01 u) and stanna-Bourgeonal (**2d**, *M*r 296.99 u) already mark the limits of perceptibility [70]; plumba-Lilial (*M*r 400.13 u), flerova-Lilial (C_13_H_20_O^285^Fl; *M*r 477.30 u) and their Bourgeonal analogues are expected to be completely odorless on the basis of their physical properties alone. The C/Si/Ge/Sn-data set offers the unique opportunity to assess the reaction of ORs to odorants in their natural environment, and by this circumvent artifacts present in *in vitro* assays.

**Table 2:** Assessment of exponential connection of odor threshold concentration [*O*] and X–C distance *r* according to Eq (2)^1^.

Compounds	*rac*-1a–*rac*-1d	2a–2d
**Fit variable *A***^2^	(0.3 ± 1.2) × 10^−1^ ng L^–1^	(2.2 ± 2.3) × 10^−4^ ng L^–1^
**Fit variable *B***^2^	2.5 ± 1.7 Å^–1^	4.2 ± 0.5 Å^–1^
**R**^**2**^	0.69	0.99

^1^X = C, Si, Ge, Sn.

^2^Error ranges denote the standard deviation.

The deviation of the Lilial series from the single receptor type odorant detection hypothesis can be understood by taking into account that we have measured a racemic (1:1) mixture of the (*R*)- and (*S*)-enantiomers. As can be seen from our docking analysis, the two enantiomers exhibit different free binding enthalpies, and therefore will interact differently with the same receptor. It might well be that one enantiomer is an agonist, while the other form is an antagonist. In this case, *rac*-**1a**–*rac*-**1d** would appear as a weak agonist, which is in line with the experimentally observed low *E*_*max*_ in comparison to **2a**–**2d** [71]. Therefore, even if only one receptor is used for odor detection, we would need two exponentials (one for each compound) for a correct description, which is not investigable with our approach. Furthermore, the odor thresholds of *rac*-**1c** and *rac*-**1d** are relatively high (Table 1, ref. [71]), so in the case of these compounds, odor detection might be coupled to another receptors, which further complicates binding analysis. With the present data set, we therefore cannot make any statement about the number of receptor types involved in the odor detection of Lilial. This result on *rac*-**1a**–*rac*-**1d** is a nice negative control for our approach. Contrary to this, compounds **2a**–**2d** exhibit a high R^2^ value of 0.98 for a single exponential fit of the *in vivo* data, which supports the hypothesis that only one receptor is necessary for the odor detection of the C/Si/Ge/Sn analogues of the Bourgeonal type. Therefore, we can assume that for the odor detection of Bourgeonal (**2a**) and its analogues **2b**–**2d**, unlike for the odor perception, only one receptor type is mostly responsible in the human nose. This coincides with the theory that only one type of OR is activating a glomerulus at threshold level (see ref. [54], Fig 2B). In the following, we make a quantitative analysis if this detection is molecule-shape based, or needs to follow a different recognition modus.

We here need to state that for the calculation of binding affinities in the form of free energies of binding, the best available methods are free energy perturbation MD calculations [36,85,86] on a fully dynamic OR/odorant complex in a membrane/solvent environment. However, the resulting error ranges are within 0.5–4.0 kcal mol^-1^, so that the differences observed in our *in vivo* data set vanish in the noise of the method. Furthermore, we do not know the exact binding mode and want to get unbiased information on the binding position. For such an approach, Docking is the right method of choice. In this context, we prefer the usage of a static protein model with minimal energy as a representative of an average protein structure, as unfavorable protein/ligand contacts will be retained at the ligand itself, and not distributed over the binding pocket and adjacent protein side chains, as it would be the case in a fully dynamic simulation. Last, we are not opting to predict exact absolute binding affinities, but the relative change of binding affinities in relation to small structural perturbations within the ligand, i.e., the elongation of the average X–C bond length (X = C, Si, Ge, Sn). This elongation will mostly affect the van der Waals radius of the XMe_3_ side chain, and thus result in different van der Waals energy terms. Unfortunately, there is no set of protein/ligand complex crystal structures available in which the C/Si/Ge/Sn switch strategy has been applied. We therefore cannot perform a benchmark on the absolute accuracy of Autodock Vina in predicting binding affinity changes in such a compound series. However, the scoring function of Autodock Vina has been explicitly parameterized to give free energies of binding as output, with a small error of 2.85 kcal mol^–1^ for the absolute binding affinities of a benchmark set of small molecules (see ref. [78]). We will not rely on a comparison of absolute binding affinities, but of differences of binding affinities induced by element substitution. By using such relative values, which come from molecules with a nearly identical scaffold, in nearly identical binding modes, as we analyze here, the absolute binding affinity errors should be removed. We therefore think that we can deduce the changes of binding affinity depending on the average X–C bond length elongation with a sufficient accuracy to make a quantitative analysis.

**Fig 4:**
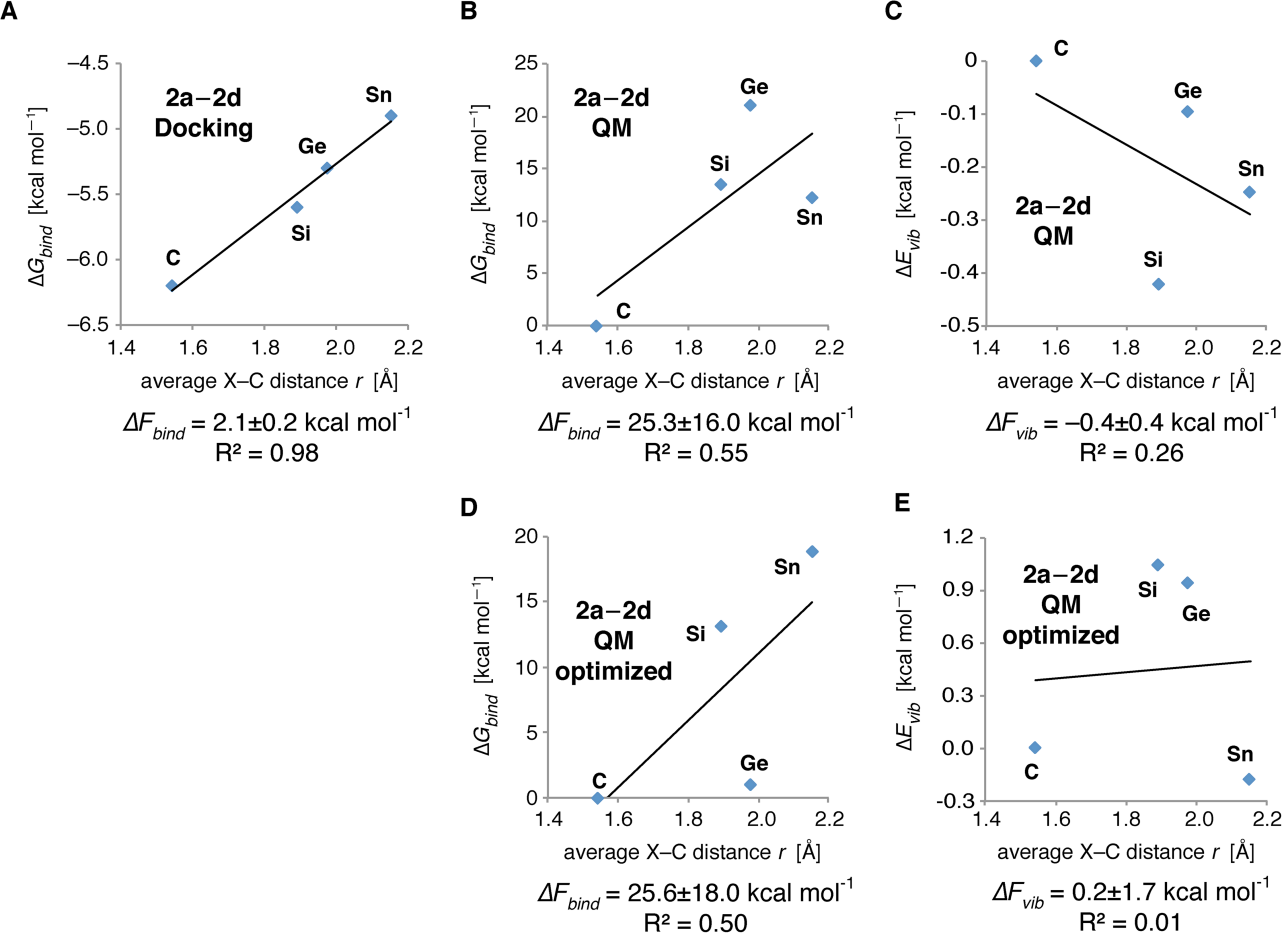
Determination of Δ*F*_*bind*_(*r*) and Δ*F*_*vib*_(*r*). Δ*F*_*bind*_(*r*) was calculated by linear regression of *ΔG*_*bind*_ from docking runs (A) or QM calculations (B) in reference to the average X–C distance *r* (X = C, Si, Ge, Sn). In a similar way, Δ*F*_*vib*_(*r*) (C) was calculated by linear regression of *ΔE*_*vib*_ from QM calculations in reference to *r*. For (B) and (C), data points are shown as the energy difference to compound **2a**. (D) and (E) display Δ*F*_*bind*_(*r*) and Δ*F*_*vib*_(*r*) after and additional ab initio QM minimization of the ligands within the rigid protein binding site.

We assume that the smelling process leads to a ligand-modulated alteration of the thermodynamic equilibrium between active and inactive receptors [87]. If the receptor activation is only related to ligand binding (which is the general paradigm for GPCRs [87]) and thus controlled by shape recognition, we can make the approximations that (i) the receptor becomes active upon ligand binding and (ii) at threshold detection conditions, the concentration of active, odorant-bound receptors [*R*_*active*_*L*_*odorant*_] is much smaller than the inactive, ligand-free receptor concentration [*R*_*inactive*_]. Accordingly, we define the activation constant *K*_*active*_ to be equal to the association constant *K*_a_ with

$$K_{active}=K_{\text{a}}=\frac{\left[R_{active}L_{odorant}\right]}{\left[R_{inactive}\right]\left[L_{odorant}\right]}=e^{-\frac{\Delta G_{bind}}{\text{R}T}}$$ (4)

with the free energy of binding Δ*G*_*bind*_ and the free ligand concentration [*L*_*odorant*_]. Furthermore, the existence of a threshold concentration implies that signaling from an olfactory neuron is coupled to a minimal number of receptors in their active state. As we only introduce small perturbations with our heteroatom exchange, we assume all investigated ligands to belong to the same pharmacological class, i.e., all to remain (full) agonists. Therefore, the minimal number of active receptors necessary for neuron signaling should not change between ligands within the Lilial and the Bourgeonal series. Last and most importantly, it was recently shown by Bush, Vasen et al. [88] that GPCR signaling in vivo is not depending on the absolute number of active receptors, but the fraction of active and inactive receptors. We therefore assume that the threshold for odor detection by one receptor type is determined by the same ratio of active and inactive receptors for all investigated ligands, so that

$$\frac{{\left[R_{active}L_{odorant}\right]}_{1}}{{\left[R_{inactive}\right]}_{1}}=\frac{{\left[R_{active}L_{odorant}\right]}_{2}}{{\left[R_{inactive}\right]}_{2}}=const.$$ (5)

with the active and odorant-bound receptor concentrations for odorant 1 and 2, [*R*_*active*_*L*_*odorant*_]_1_ and [*R*_*active*_*L*_*odorant*_]_2_, and the respective inactive, ligand-free receptor concentrations [*R*_*inactive*_]_1_ and [*R*_*inactive*_]_2_. As we observe in the docking analysis, Δ*G*_*bind*_ is indeed negatively linearly coupled with the average X–C distance *r* (cf. Fig 4) obtained from QM calculations, which is in line with our initial assumptions formulating Eq (2). Eq (4) furthermore implies that *K*_a_ is coupled to the odor threshold concentration [*O*]: under the conditions of odor threshold determination, the free odorant concentration [*L*_*odorant*_] close to the receptor equals the concentration of the odorant in the nasal cavity, which again is equal to the odorant concentration in the air volume leaving the sniffing port. At the point of odor detection, [*L*_*odorant*_] is therefore equal to [*O*]. For the two odor thresholds of ligands 1 and 2, [*O*]_1_ and [*O*]_2_, connected with two X–C distances *r*_1_ and *r*_2_, we can use Eq (2) to formulate that

$$\frac{{\left[O\right]}_{2}}{{\left[O\right]}_{1}}=e^{B\cdot (r_{2}-r_{1})}=e^{B\cdot \Delta r}=e^{-\frac{\Delta \Delta G_{threshold}}{\text{R}T}},$$ (6)

with the *r*-dependent threshold free energy *ΔΔG*_*threshold*_. Analogously, for the association constants of the two ligands, *K*_a,1_ and *K*_a,2_, we can formulate that

$$\frac{K_{\text{a},2}}{K_{\text{a},1}}=e^{-\frac{\Delta \Delta G_{\mathrm{bind}}}{\text{R}T}}$$ (7)

with

$$\Delta \Delta G_{\mathrm{bind}}=\Delta G_{\mathrm{bind},2}-\Delta G_{\mathrm{bind},1}.$$ (8)

With Eq (4) we can state that

$$e^{-\frac{\Delta \Delta G_{bind}}{\text{R}T}}=\frac{K_{\text{a},2}}{K_{\text{a},1}}=\frac{{\left[R_{active}L_{odorant}\right]}_{2}{\left[R_{inactive}\right]}_{1}{\left[L_{odorant}\right]}_{1}}{{\left[R_{inactive}\right]}_{2}{\left[L_{odorant}\right]}_{2}{\left[R_{active}L_{odorant}\right]}_{1}}.$$ (9)

As mentioned above, under odor threshold determination conditions, the concentration of the free unbound ligand [*L*_*odorant*_] is equal to the measured threshold concentration [*O*], so

$$e^{-\frac{\Delta \Delta G_{bind}}{\text{R}T}}=\frac{{\left[R_{active}L_{odorant}\right]}_{2}{\left[R_{inactive}\right]}_{1}{\left[O\right]}_{1}}{{\left[R_{inactive}\right]}_{2}{\left[O\right]}_{2}{\left[R_{active}L_{odorant}\right]}_{1}}.$$ (10)

Last, with Eq (5) we can connect *ΔΔG*_*threshold*_ and *ΔΔG*_*bind*_ by

$$e^{-\frac{\Delta \Delta G_{bind}}{\text{R}T}}=\frac{{\left[O\right]}_{1}}{{\left[O\right]}_{2}}=e^{\frac{\Delta \Delta G_{threshold}}{\text{R}T}}$$ (11)

and thus

$$\Delta \Delta G_{\mathrm{bind}}=-\Delta \Delta G_{\mathrm{threshold}}$$ (12)

With this, we can define the *r*-depending change of the odor threshold factor *ΔF*_*threshold*_(r) with

$$\Delta F_{threshold}\left(r\right)=\frac{\Delta \Delta G_{threshold}}{\Delta r}=\frac{dG_{threshold}}{dr}=-\text{RT}\cdot B$$ (13)

and analogously the *r*-depending binding factor *ΔF*_*bind*_(r) with

$$\Delta F_{bind}\left(r\right)=\frac{\Delta \Delta G_{bind}}{\Delta r}=\frac{dG_{bind}}{dr},$$ (14)

which both are factors with the dimension of a force. While we can calculate *ΔF*_*threshold*_(r) from the single exponential fit of our experimental *in vivo* data (see Table 2), we obtain *ΔF*_*bind*_(r) from our docking investigations summarized in Fig 4A and Table 1.

Here, we have to state again that like in the case of the *in vitro* experimental data, the *in vivo* threshold data contain an unknown amount of ambiguity due to unknown and potentially non-linear effects during signal processing in the olfactory neuronal cells, the olfactory bulb, or the human brain. Similar to our approach on the computational side, where we use the relative variable *ΔF*_*bind*_(*r*) to cancel out absolute errors, we here now use the relative variable *ΔF*_*threshold*_(*r*) to cancel out these non-linear effects. *ΔF*_*threshold*_(*r*) therefore should only be depending on changes in *r*, and not on any other effects during signal processing in the human body. Combining Eqs (12), (13) and (14), we finally yield

$$-\Delta F_{threshold}\left(r\right)=\Delta F_{bind}\left(r\right).$$ (15)

with the *r*-dependent free odor threshold energy factor *ΔF*_*threshold*_(*r*) and the *r*-dependent free binding energy factor *ΔF*_*bind*_(*r*). We can now formulate the Null hypothesis that if odor detection in the nose is only dependent on ligand/binding pocket and electrostatics complementarity, then Eq (15) must be fulfilled.

At physiological temperature, i.e. 310 K, the experimentally determined *ΔF*_*threshold*_(*r*) is –2.6 ± 0.3 kcal mol^–1^ Å^–1^. From a linear regression of the computationally determined X–C distances for compounds **2a**–**2d** and the respective *ΔG*_*bind*_ values in Table 1, we can calculate that *ΔF*_*bind*_(*r*) = 2.1 ± 0.2 kcal mol^–1^ Å^–1^ for Bourgeonal, as can be seen from Fig 4A. As both values overlap in their standard deviations and thus follow Eq (15), we state that our Null hypothesis is true and the odor detection of Bourgeonal (**2a**) and its derivatives **2b**–**2d** is mainly based on the recognition of the molecular shape (as we defined it in the introduction) and matching electrostatics in the human nose.

As these results are dependent on the accuracy of the Autodock scoring function, which is not suited to describe van der Waals interactions of the ligand heteroatoms used in this approach with the protein, we use QM calculations on the complex structures obtained from Docking to estimate *ΔF*_*bind*_(*r*) [79]. The results, displayed in Fig 4B, exhibit a higher value of *ΔF*_*bind*_(*r*) = 25.3 ± 16.0 kcal mol^–1^ Å^–1^. However, the linear fit of the data is highly unreliable (R^2^ = 0.55), so that we only can state that we see a qualitative match of our results from Docking and QM calculations. To compensate for possible errors coming from the Docking poses themselves, we carried out an *ab initio* optimization of the ligands in the rigid binding pocket model. As Fig 4D shows, the resulting *ΔF*_*bind*_(*r*) = 25.6 ± 18.0 kcal mol^–1^ Å^–1^ and R^2^ = 0.50 are nearly identical to the values for the Docking poses themselves, showing the robustness of our approach. The Autodock scoring function was parameterized with based on the PDBbind data set [89], i.e. a set of protein/ligand crystal structures and their experimentally determined binding free energies. It therefore implicitly takes into account changes in protein and ligand solvation. The QM determination of *ΔG*_*bind*_ is based on the calculation of the partition function including electronic, translational, rotational, and vibrational contributions. It thus misses the contributions from solvation. Besides this, we rate the Docking results as reliable, as the Docking procedure and the generation of the homology model both utilize principles of Molecular Mechanics (MM). That means: The homology model was built and minimized using the united atom AMBER forcefield implemented in MOBY. Furthermore, in Docking, the protein and ligand topologies again are based on a united atom treatment, as well. Switching now over to a QM assessment of the ligand binding modes within the AMBER based binding site, we encounter several difficulties: first of all, we need to include all hydrogen atoms, and thus change the van der Waals surface within the binding site and of the ligand. Second, this introduction induces a large number of weak dipoles, which are not present in the case of a united atom scheme (atomic charges of aliphatic carbon/hydrogen united atoms = 0) and the scoring function of Vina. Third and last, under QM treatment, the side chain conformation distribution would most likely differ from the Molecular Mechanics case, so that we would need to perform a QM optimization of the homology model, which is out of the question. Therefore, especially due to the presence of additional Coulomb interactions within the binding site, the QM binding energies should be more sensitive to changes in atom distances within the binding site, which is exactly what we observe. However, as stated above, the binding site model was not created in a QM environment. We therefore hold the results from Docking including solvation contributions to be more reliable, and we only use the QM results as an additional counter check if its trend agrees with the MM results. However, this type of calculation allows us to assess the difference in vibrational energies between the ligands in the binding pocket.

The vibrational theory states that OR activation is caused by vibration assisted electron transfer [41,45,47]. The major benefit of this theory was that it could explain minor changes in odor perception upon H/D exchange [43,44]. The vibrational theory proposes that the G protein is connected to the olfactory receptor via a disulphide bond, and an electron transfer from an extracellular NADPH molecule to this bond causes disulphide reduction, followed by bond breaking and G protein off-diffusion. There are two ways how such a transfer might happen: the first possibility is that the necessary transfer energy comes from the vibrational energy component Δ*E*_*vib*_ in the ligand Hamiltonian. Fig 4C displays the respective *ΔF*_*vib*_(*r*), which we calculated as

$$\Delta F_{vib}\left(r\right)=\frac{\Delta \Delta E_{vib}}{\Delta r}=\frac{dE_{vib}}{dr}.$$ (16)

We observe that *ΔF*_*vib*_(*r*) = 0.4 ± 0.4 kcal mol^–1^ Å^–1^ with an R^2^ = 0.26. We therefore can make that statement that we do not see any relation between vibrational energies and changes in X–C distance *r*, and thus do not see a correlation between odorant vibration and physiological odorant detection. Furthermore, *ΔF*_*vib*_(*r*) is significantly smaller than the experimentally observed *ΔF*_*threshold*_(*r*). That means, the vibrational energy difference within our ligand series is too small to explain the differences in odor thresholds.

The second possibility is a vibration-assisted inelastic electron tunneling as was proposed by Turin in ref. [41]. Here, the potential gap *U* between the extracellular NADPH and the intracellular receptor / G protein disulphide bond is bridged by a vibration of an odorant molecule bound to the receptor, which allows electron tunneling between donor and acceptor for disulphide reduction. According to references [90–92], this effect can be caused by any vibration with an energy *E*_*vib*_ such that *E*_*vib*_*/e* ≤ *U*, with an energy range *eU*–*E*_*vib*_ ≤ [0.025; 0.05] eV.

That is: all vibrations with an energy *eU*–*E*_*vib*_ ≤ [0.025; 0.05] eV will facilitate inelastic electron tunneling, and thus lead to receptor activation.

The crucial point here is: What is the biologically possible range of *U*? Using tabulated values for the respective half-cell potentials (NADPH + H^+^⇔ NADP^+^ + 2H^+^ + 2e^-^: 0.32 V [93,94]; RSSR + 2H^+^ + 2e^-^ ⇔ 2 RSH: -0.22 to -0.29 V [95], we obtain a range of *U* = 0.1 to 0.03 V, and thus *E*_*vib*_ = 0.1 to 0.03 eV. As a more realistic fixpoint, we use the half-cell potential for glutathione of -0.26 V [95], as it contains a prototypical cysteine disulphide bond which should occur between receptor and G protein. In this specific case, *U(Glutathione)* = 0.06 V, and *eU(Glutathione)* = 0.06 eV, respectively.

Translated into wavenumbers: the range of tunneling facilitating vibrations determined by *eU*–*E*_*vib*_ is equal to 200–400 cm^-1^; the upper limit for tunneling facilitating vibrations determined by *eU* is about 240–800 cm^-1^; and the upper limit for tunneling facilitating vibrations for a cysteine disulphide bond as donor is approximately 480 cm^-1^. In other words: if the vibration theory would be true, then compounds exhibiting vibrations between 80 cm^-1^ and 480 cm^-1^ should activate an olfactory receptor with a prototypical cysteine disulphide bond. If the disulphide bond is modulated in its energy level by unknown means to the upper limit given in [95], this range can be extended to 400 cm^-1^ to 800 cm^-1^.

As we calculated the vibrational frequencies of our investigated ligands, we can compare these frequencies, the experimentally determined activation patterns, and the activation patterns predicted by the vibration theory with each other. The only differences in vibrations found by us were X–C bend vibrations, and combined symmetric X–C side chain vibrations. Table 3 gives an overview over these two types of vibrations in the Bourgeonal compound series **2a–2d**.

**Table 3:** C-X vibrations in compounds 2a–2d.

Compounds	C–X bend vibrations (cm^-1^)		symmetric C-X side chain vibrations (cm^-1^)
	calculated	IR measurements^1^	calculated
**2a**	1130	1109	675
**2b**	883	840	606
**2c**	847	820	554
**2d**	797	776	489

^1^see ref.[71].

We now analyze the two discussed frequency ranges that might lead to activation under the vibration theory:

400–800 cm^-1^ (upper limit model): **2d** should be most active, as it exhibits two vibrations in the activation range (797 cm^-1^ and 489 cm^-1^). Furthermore, **2a–2c** should all be active (vibrations at 675 cm^-1^, 606 cm^-1^, and 554 cm^-1^, respectively).

80–480 cm^-1^ (Glutathione model): **2d** should be the only active compound (vibration at 489 cm^-1^). Furthermore, **2a–2c** should be completely inactive.

Neither of the two models is in agreement with the experimentally derived data from our earlier works [71]. Therefore, the “vibration theory” is at odds with experimental observations. In addition, the frequency ranges calculated by us that should lead to G protein activation are far from the range of C–H and C–D vibrations [96]. Taken together, the “vibration theory” is at odds with our own data, and even cannot explain results from H/D exchange experiments.

We are aware that a molecular shape based recognition alone cannot explain H/D experimental data, either. However, some of us authors have recently proposed an activation mechanism based on matching protein/ligand dynamics [49,50]. Here, in the case of highly flexible ligands, besides a molecular shape matching the ligand binding site, it is matching dynamics between the highly flexible ligand and residues forming the binding site that determines a successful receptor activation. A H/D exchange is not only altering vibrational properties, but the molecular weight, as well. At a comparable temperature, and thus comparable mean kinetic energy, this results in a reduced molecular velocity of deuterated compounds in comparison to their hydrogen counterparts. Therefore, this theory of matching protein/ligand dynamics is capable to extend a molecular shape recognition to explain changes in odorant recognition upon H/D exchange. However, a verification of this hypothesis will be a work of its own.

Therefore, we can state that we see no evidence for vibrational ligand recognition in hOR17-4. Our result is in good agreement with the findings of Block et al. from theoretical and *in vitro* analysis of the interaction of isotopically labeled odorants with a set of different olfactory receptors [97], which we now extend to an analysis of interaction in vivo. Furthermore, our results agree well with the findings on thiol recognition by the human olfactory receptor hOR2T11 [37–40], which only responses to low molecular weight thiols with one to four carbons; thiols with more carbon atoms and alcohols give no response. This discovery is at variance with the vibrational recognition which claims that detection of thiols is associated with the S–H vibrational band [41].

Combining experimental and theoretical results, while we cannot make a statement on the odorant interaction / detection mechanism for Lilial (*rac*-**1a**), we can conclude that the molecular shape and electrostatics are the major parameters for the detection of Bourgeonal (**2a**) in the human nose. This statement is based on a highly selective structure–affinity assay: Bourgeonal (**2a**) and its derivatives **2b**–**2d** share a high degree of structural similarity and exhibit very small and defined differences in a single molecular fragment. Again, we have to be aware that the *in vivo* data points exhibit a high standard deviation. However, this data set represents the state-of-art in qualitative in the human nose, and allows us the quantitative analysis of the native detection of compounds **2a**–**2d**. We could show that a single olfactory receptor type most likely mediates *in vivo* odor detection, while multiple receptors are required for odor perception by pattern recognition process in mammals. While we assume this receptor type to be hOR17-4, which we used in our *in vitro* assays, we cannot ensure it completely. While the experimental *in vitro* thresholds (i.e., the observable onset of the sigmoid ligand concentration / cell response curve, see Ref. [71], Fig **1B**) for Lilial and Bourgeonal compounds are in the μM range, the measured *in vivo* thresholds are in the lower pM range of odorant air concentration (see Ref. [71], Tab. 1). However, this discrepancy might be compensated by periperception processes, i.e. raising the local odorant concentration close to the receptors via pre-binding to the nasal mucus or odorant-binding proteins [66,67]. Furthermore, we have a convincing agreement of the theoretical data coming from docking into the hOR17-4 model, and the experimental threshold data. We therefore think that either hOR17-4, or a different single OR type with a similar binding pocket performs odorant detection of compounds **2a**–**2d** in the human nose. Last, we do not see any connection between odorant / binding pocket vibrational energies and the physiologic odor threshold, meaning that there is no contribution from odorant vibrations to olfactory receptor activation.

## Conclusions

In Summary, a QSAR analysis based on the *in vivo* odor threshold determination and *in vitro* assays allowed us to differentiate between the discussed mechanisms for odorant recognition for the example of the olfactory receptor hOR17-4. As demonstrated by our comparison of theoretical and *in vivo* data exemplarily for Bourgeonal, odorant recognition under threshold conditions *in vivo* is mainly based on the molecular shape, i.e. complementary van der Waals surfaces, and matching electrostatics of the odorants, and not on odorant vibrations. Furthermore, we showed that a single olfactory receptor type is responsible for odor detection of Bourgeonal in the human nose, which we attribute to hOR17-4. These results demonstrate that it is indeed the shape and electronic surface structure that determines the interaction of an odorant with its ORs, at least in the case of hOR17-4. The C/Si/Ge/Sn switch strategy [68–71] used for these studies proved to be a powerful tool to provide insight into the molecular mechanism of odorant recognition. Our combined experimental and theoretical approach can be used to elucidate the odor detection mechanism of odorants of interest directly *in vivo*, and might further be extended to gain insight into odorant discrimination mechanisms as well.

## Supporting information

S1 File Docking results for Lilial compounds 1a – 1d. Contains: Pymol PSE file with Docking structures, and *.log files with the respective free energies of binding. Naming: “Lil” = compounds (*S*)-**1a – 1d**; “Lilinv” = compounds (*R*)-**1a – 1d**.
(ZIP)

S2 File Docking results for Bourgeonal compounds 2a – 2d. Contains: Pymol PSE file with Docking structures, and *.log files with the respective free energies of binding.
(ZIP)

S3 File QM data on free energies and vibrational energies for Bourgeonal compounds 2a – 2d. Contains: Gaussian output files. File naming: “C”: compound **2a**; “Si”: **2b**; “Ge”: **2c**; “Sn”: **2d**. “fix” for calculation of fixed ligand, “ligfree” for free minimization of a ligand in the binding pocket.
(ZIP)
